# Genetic engineering of medium-chain-length fatty acid synthesis in *Dunaliella tertiolecta* for improved biodiesel production

**DOI:** 10.1007/s10811-017-1210-7

**Published:** 2017-07-15

**Authors:** Huixin Lin, Yuan Kun Lee

**Affiliations:** Department of Microbiology and Immunology, Yong Loo Lin School of Medicine, National University of Singapore, Block MD4, 5 Science Drive 2, Singapore, 117545 Singapore

**Keywords:** Microalgae, Thioesterase, Ketoacyl-ACP synthase, Lipid, Fatty acid

## Abstract

Genetic engineering of microalgae to accumulate high levels of medium-chain-length fatty acids (MCFAs) represents an attractive strategy to improve the quality of microalgae-based biodiesel, but it has thus far been least successful. We demonstrate that one limitation is the availability of fatty acyl-acyl carrier protein (ACP) substrate pool for acyl-ACP thioesterase (TE). A combinational expression platform that involved plant lauric acid-biased TE (C12TE) and MCFA-specific ketoacyl-ACP synthase (KASIV) increased lauric acid (C12:0) and myristic acid (C14:0) accumulation by almost sevenfold and fourfold, respectively, compared with native strain. These findings suggest a platform for further investigation into the enlargement of MCFA acyl-ACP substrate pool as an approach to sustainably improve quality of microalgae-based biodiesel with regard to MCFA production.

## Introduction

In response to the world energy crisis and the global climate change, microalgae-based biodiesel has attracted renewed interest in an effort to search for sustainable development (Zinoviev et al. 2010). While most of the contemporary microalgae-based biodiesel research efforts focus on improving biomass accumulation and lipid productivity, relatively few studies have investigated the quality of microalgae oil. The fuel performance properties of the biodiesel are directly dependent on the structural features of the biologically derived fatty acid (FA) profiles. Microalgal oils typically contain longer FA carbon chain lengths and higher degrees of unsaturation compared to regular petroleum diesel (Stansell et al. 2012). These biomass feedstock traits lead to poor cold flow temperature properties (longer FA carbon chain lengths) and oxidative instability (higher degrees of unsaturation) (Knothe et al. 2005).

Genetic engineering of FA profiles in microalgae to synthesize medium-chain-length FAs (MCFAs), from C8 to C14, is preferable for production of biodiesel (Durrett et al. 2008). The chain lengths of FAs are determined by acyl-acyl carrier protein (ACP) thioesterases (TEs). TEs are highly specific and are classified according to their substrate preferences, namely FatA and FatB. FatA class enzymes prefer C18:1-ACP as a substrate, whereas FatB class enzymes prefer saturated acyl-ACPs (Jing et al. 2011). Certain plant species accumulate MCFAs in their seed oil storage, and accordingly, there exist chain-length specific TEs that prematurely cleave the corresponding FAs from the growing fatty acyl-ACPs (Voelker and Kinney 2001). For example, *Umbellularia californica* (Davies et al. 1991) and *Cuphea hookeriana* (Dehesh et al. 1996) seeds accumulate up to 90% MCFAs in the triacylglycerols (TAGs). The chain-length specific TEs were isolated and identified in both species as the cause of the unusual accumulation (Thelen and Ohlrogge 2002). The first proof of principle of introducing chain-length specific TE to alter FA profile was demonstrated by Voelker and colleagues (1992) via engineering a lauric acid (C12:0)-biased FatB TE from *U. californica* into *Arabidopsis thaliana* for a 24% increase in C12:0 production.

Divergent TEs with substrate specificities for FAs with chain lengths less than C16 were previously engineered to alter FA contents in microalgae. C12:0-biased FatB TE from *U. californica* and myristic acid (C14:0)-biased FatB TE from *Cinnamomum camphora* were transformed into *Phaeodactylum tricornutum*, which redirected FA synthesis to, albeit at low yield (6% increase in C12:0 and 15% increase in C14:0), the desired medium-chain-length phenotype (Radakovits et al. 2011). Similar plant TEs were introduced into *Chlamydomonas reinhardtii*, but no change in FA profile was observed (Blatti et al. 2012). Distinct TEs in microalgae (*C. reinhardtii* and *P. tricornutum*) have been identified, and they are plant FatA/FatB hybrid TEs, demonstrating promiscuity in FA substrates they act upon (Gong et al. 2011; Blatti et al. 2012). Overexpression of the native *C. reinhardtii* TE resulted in a corresponding phenotype of enhanced MCFA production (Blatti et al. 2012). On the other hand, overexpression of *P. tricornutum* TE did not result in altered FA composition in *P. tricornutum* (Gong et al. 2011).

Past efforts to increase MCFA production in microalgae by genetic modifications of chain-length specific TEs have met with limited success. We are interested to investigate a less considered mechanism that may limit MCFA accumulation, and which is the availability of fatty acyl-ACP substrate pool for the TE activity. Such a mechanism may be related to the intrinsic competitive nature of endogenous TE whereby broadly specific microalgae TE could outrival chain-length specific exogenous TE in the hydrolytic activity of fatty acyl-ACPs. This control is likely related to the functions of lipids in the structural membrane compositions of unicellular microalgae, with primarily long-chain-length FAs (LCFAs), from C16 to C18 (Tang et al. 2011). Although TEs are necessary determinants of MCFA phenotype, other candidates for chain-length regulation activities may exist and they are ketoacyl-ACP synthases (KASs), the condensing enzymes responsible for the cyclic two carbon elongations in FA synthesis (Dehesh 2001). We speculate that a condensing enzyme has the ability to proportionate different chain lengths of fatty acyl-ACP substrates for the hydrolysis by TEs. In this study, we describe the results of various genetic modification approaches to increase MCFA production in *Dunaliella tertiolecta*. Our results additionally address whether endogenous TE and/or MCFA-specific KAS are involved in MCFA accumulation.

## Materials and methods

### Microalgae strains and culture conditions

Strain LB-999 of *D. tertiolecta* was obtained from the UTEX Culture Collection of Algae (University of Texas, Austin, TX, USA). *Dunaliella tertiolecta* cells were grown in a batch system in a sterile ATCC-1174 liquid medium (American Type Culture Collection, Manassas, VA, USA) containing 0.5 M NaCl on a rotary shaker at 25 °C, under a 14 h light/10 h dark regime (50 μmol photons m^−2^ s^−1^). Cell densities were measured using an automatic cell counter (Bio-Rad Laboratories, USA). For the purpose of clarity, all experiments were performed in independent biological/technical triplicates and began at equal cell densities for standardization unless otherwise stated.

### Plasmid construction

**Fig. 1:**
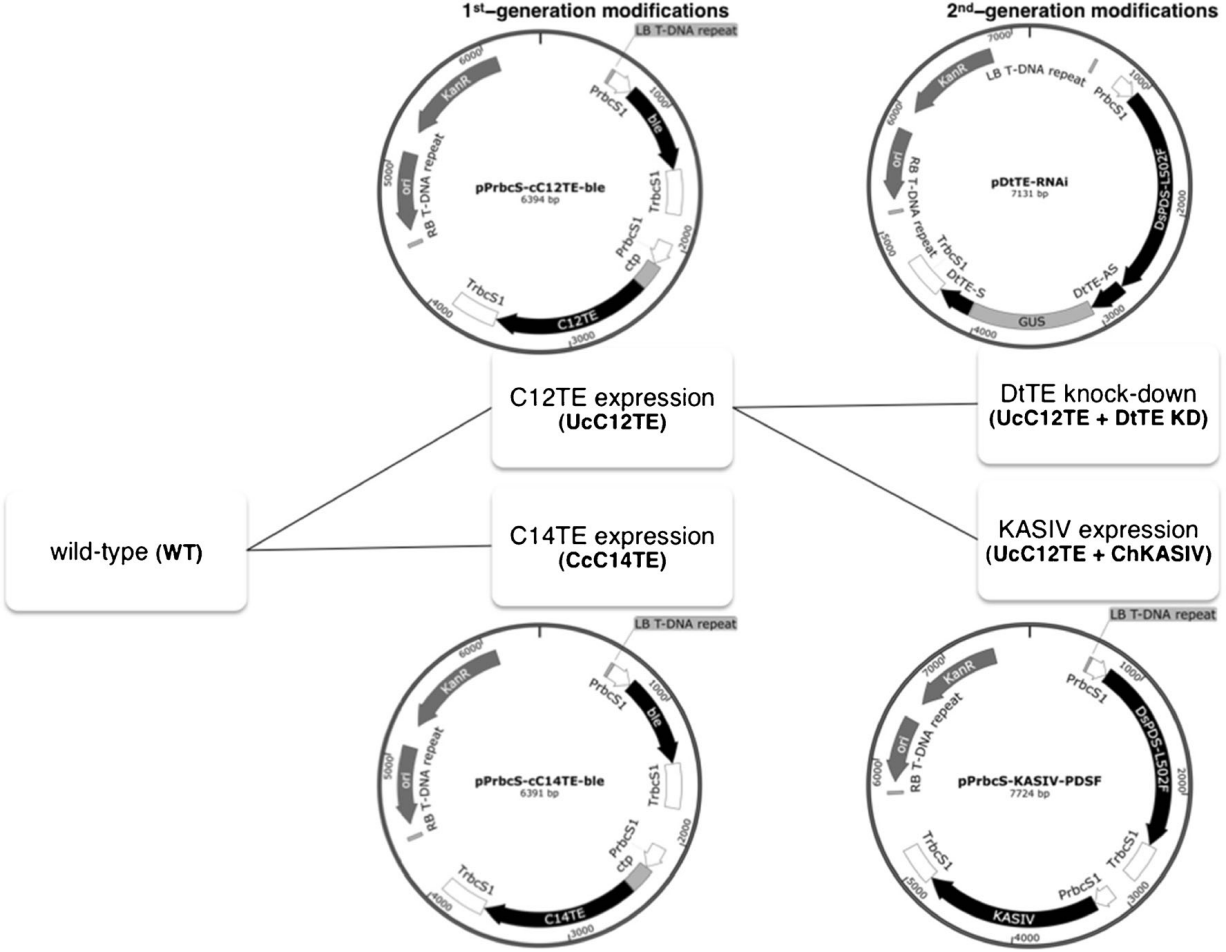
Genetic engineering of *D. tertiolecta* to increase MCFA production. Sequential modifications are shown in flow chart, and detailed modifications are represented in schematic diagrams of transformation plasmids. As shown for pPrbcS-cC12TE-ble and pPrbcS-cC14TE-ble, *U. californica* C12:0-biased FatB TE (*C12TE*) and *C. camphora* C14:0-biased FatB TE (*C14TE*), together with chloroplast transit peptide (*ctp*), are respectively flanked with *D. tertiolecta* ribulose bisphosphate carboxylase small subunit 1 (*DtrbcS1*) promoter and terminator. Plasmid backbone contains kanamycin resistance cassette and positively selectable marker zeocin (ble) controlled by the DtrbcS1 promoter. In pDtTE-RNAi, the hairpin loop was generated using *D. tertiolecta* TE (*DtTE*) anti-sense (*DtTE-AS*) and sense (*DtTE-S*) fragments linked with β-glucuronidase (*GUS*). In pPrbcS-KASIV-PDSF, *Cuphea hookeriana* MCFA-specific KAS (*KASIV*), together with ctp, are flanked with the DtrbcS1 promoter and terminator. Plasmid backbone contains kanamycin resistance cassette and positively selectable marker norflurazon (*DsPDS-L502F*) controlled by the DtrbcS1 promoter

The plasmids used in this study are listed in Fig. 1. All of the TEs and KAS were synthesized and codon optimized for the expression in *D. tertiolecta* (GenScript Corporation, USA). The TE sequences used in this study were derived from a lauric acid-biased TE (C12TE) from *U. californica* (GenBank U17097.1) and a myristic acid-biased TE (C14TE) from *C. camphora* (GenBank U31813.1) (Radakovits et al. 2011; Blatti et al. 2012). The MCFA-specific KAS (KASIV) was derived from *C. hookeriana* (GenBank AAC68861.1) (Dehesh et al. 1998). Each gene, including a chloroplast-targeting sequence from *D. tertiolecta* sedoheptulose-bisphosphatase (DtSBPase) (GenBank KF193066), was cloned into a pGreenII 0000 plasmid (Hellens et al. 2000) that contained either zeocin (*ble*) (Stevens et al. 1996) or norflurazon (*DsPDS-L502F*) (Liu et al. 2013) conferring resistance genes, under the control of *D. tertiolecta* ribulose bisphosphate carboxylase small subunit 1 (DtrbcS1) promoter (Walker et al. 2005). The overexpression plasmids were abbreviated as pPrbcS-cC12TE-ble, pPrbcS-cC14TE-ble, and pPrbcS-KASIV-PDSF. A 300-bp fragment of *D. tertiolecta* TE (DtTE) (GenBank KX815261) was used to construct the DtTE knockdown plasmid, pDtTE-RNAi, driven by the DtrbcS1 promoter, according to our previous study (Lin et al. 2013).

### Microalgae transformation

pPrbcS-cC12TE-ble, pPrbcS-cC14TE-ble, pDtTE-RNAi, and pPrbcS-KASIV-PDSF were transformed into wild-type and mutant cC12TE_A4 (from UcC12TE background) *D. tertiolecta* (Fig. 1) using the glass beads method (Lin et al. 2013). Selection of transgenic *D. tertiolecta* strains was done in either 8 μg mL^−1^ zeocin (Invitrogen, USA) or 0.4 μg mL^−1^ norflurazon (Sigma-Aldrich, USA).

### Screening of transgenic strains

Genomic DNA of native and transgenic *D. tertiolecta* cells was extracted using a modified Scott Newman’s method (Newman et al. 1990) and used as a template for genomic PCR analysis to confirm the existence of transgenes. The gene-specific primers used for the detection of transformation plasmids are listed in Table 1. Each PCR reaction, consisting of 1 μg genomic DNA, was set up according to the manufacturer’s instructions (Thermo Fisher Scientific, USA). Amplification was performed at 95 °C for 5 min and cycled for 30 rounds of denaturation (95 °C for 30 s), annealing (58 °C for 30 s), and extension (72 °C for 30 s), followed by a final extension (72 °C for 5 min).

**Table 1:** List of primers used for detecting various genes in this study

Primer name	Primer sequence (5′–3′)
C12TE-GPCR-F	AGAAGCAGTGGACCAACCTG
C12TE-GPCR-R	AGCGGTGCTATCGTTGAACT
C14TE-GPCR-F	ACGGCACCAAGTTCAGCTAC
C14TE-GPCR-R	CTCCTGGTCCTGGTGTTCAT
DtTE-AS1-GPCR-F	ACGTGCTGGTGGCAGAACCA
DtTE-GUS-GPCR-R	TGCCATGTTCATCTGCCCAG
KASIV-GPCR-F	GCGAGATCAAGAGCTTCTCC
KASIV-GPCR-R	CTTGTTCAGCTCCTTCATGG
C12TE-RTF	AACCAGCACGTGAACAACAT
C12TE-RTR	GGTGCACTCCCTCCTGTACT
C14TE-RTF	GACGAGGAGATCAAGAAGCC
C14TE-RTR	ATGTTGTTCACGTGCTGGTT
DtTE-RTF	GGGACATGGTCACAGTTGAG
DtTE-RTR	GGTGGCAGAACCATACTCCT
KASIV-RTF	GCGAGATCAAGAGCTTCTCC
KASIV-RTR	CTTGTTCAGCTCCTTCATGG
DtTUB-RTF	CAGATGTGGGATGCCAAGAACAT
DtTUB-RTR	GTTCAGCATCTGCTCATCCACCT

### Real-time quantitative PCR

Total RNA was extracted from native and transgenic *D. tertiolecta* cells with the RNeasy Plant Mini Kit (Qiagen, USA) and reverse transcribed to random-primed complementary DNA (cDNA) with the SuperScript III First-Strand Synthesis System (Invitrogen) according to the manufacturer’s instructions. The real-time quantitative PCR (qPCR) was performed using a Bio-Rad CFX 96 Touch qPCR Detection System (Bio-Rad Laboratories) and 2× Maxima SYBR Green/ROX qPCR Master Mix (Thermo Fisher Scientific) according to the manufacturer’s instructions. Specific primers used for the analysis of gene expression are listed in Table 1. *D. tertiolecta* beta-tubulin gene, *DtTUB*, was used as an internal standard control. To normalize the relative gene expression across all samples, the starting amount of cDNA was standardized for all the samples.

### FA profiling and neutral lipid quantification

The FA composition of lipids was analyzed using direct acid-catalyzed derivatization of FA methyl esters (FAMEs) as described previously (Lee et al. 2014) with minor modifications. The lyophilized sample was treated with 0.2 mL chloroform/methanol (2:1, *v*/*v*) and 0.3 mL of 1.25 M methanolic hydrochloride for 1 h at 85 °C. Tridecanoic acid (Sigma-Aldrich) was included to correct for the loss of FAMEs from incomplete derivatization and subsequent extraction. Then, 1 mL hexane was used to extract the derivatized FAMEs for 1 h at room temperature. Extracted FAMEs were analyzed using an Agilent 7890B gas chromatograph (GC) (Agilent Technologies, USA) equipped with an Agilent 5977A electron ionization mass spectrometric detector (MS). The GC inlet temperature was set to 240 °C with an injection volume of 1.0 μL and a 5:1 split ratio. Helium was used as the carrier gas in a constant flow rate at 1.0 mL min^−1^. A highly polar stationary phase-fused silica capillary column of cyanopropyl polysiloxane with a film thickness dimension of 100 m × 0.25 mm × 0.20 μm (CP-Sil 88; Agilent Technologies) was used for analysis. The GC parameters were programmed as follows: the initial oven temperature of 80 °C was held for 3 min before being ramped at 4 °C min^−1^ to 220 °C for 5 min and at 4 °C min^−1^ to 240 °C for 10 min. The total run time was 55 min. The MS parameters were set as follows: the mass range was 40 to 500 Da in full scan mode, and the auxiliary, MS source, and MS quad temperatures were set to 240, 230, and 150 °C, respectively. Quantification of FAMEs was performed using the MassHunter Workstation software (Agilent Technologies) against a series of C4–C24 FAME standards (Sigma-Aldrich). The FA composition in *D. tertiolecta* indicated the percentage composition of each FA obtained by dividing the quantity of each FA by the sum of total FAs normalized to sample dry weight and FAME recovery. Neutral lipids were quantified by a Nile red staining method (Tan et al. 2016).

## Results and discussion

### Generation of MCFA producing *D. tertiolecta* strains

**Fig. 2:**
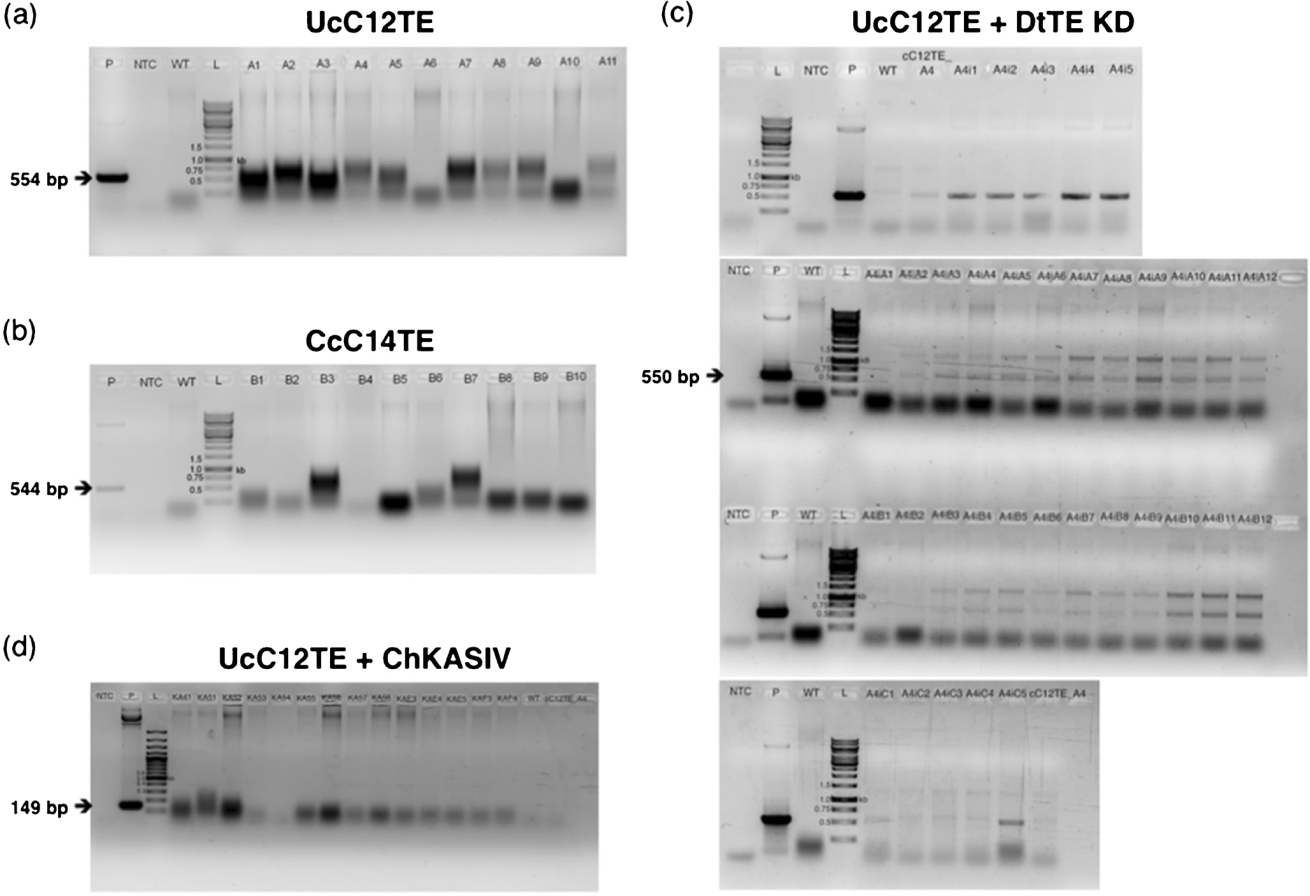
Genomic PCR identification of MCFA producing *D. tertiolecta* strains. Amplification of the partial fragments of transgenes from the genomic DNA of mutant and wild-type *D. tertiolecta* cells using gene-specific primers. *P* plasmid, *NTC* non-template control, *WT* genomic DNA wild-type, *L* 1-kb ladder marker. **a** Genomic DNA pPrbcS-cC12TE-ble mutants. **b** Genomic DNA pPrbcS-cC14TE-ble mutants. **c** Genomic DNA pDtTE-RNAi mutants. **d** Genomic DNA pPrbcS-KASIV-PDSF mutants. *UcC12TE U. californica* C12:0-biased FatB TE, *CcC14TE C. camphora* C14:0-biased FatB TE, *DtTE KD D. tertiolecta* TE knockdown, *ChKASIV C. hookeriana* MCFA-specific KAS

**Fig. 3:**
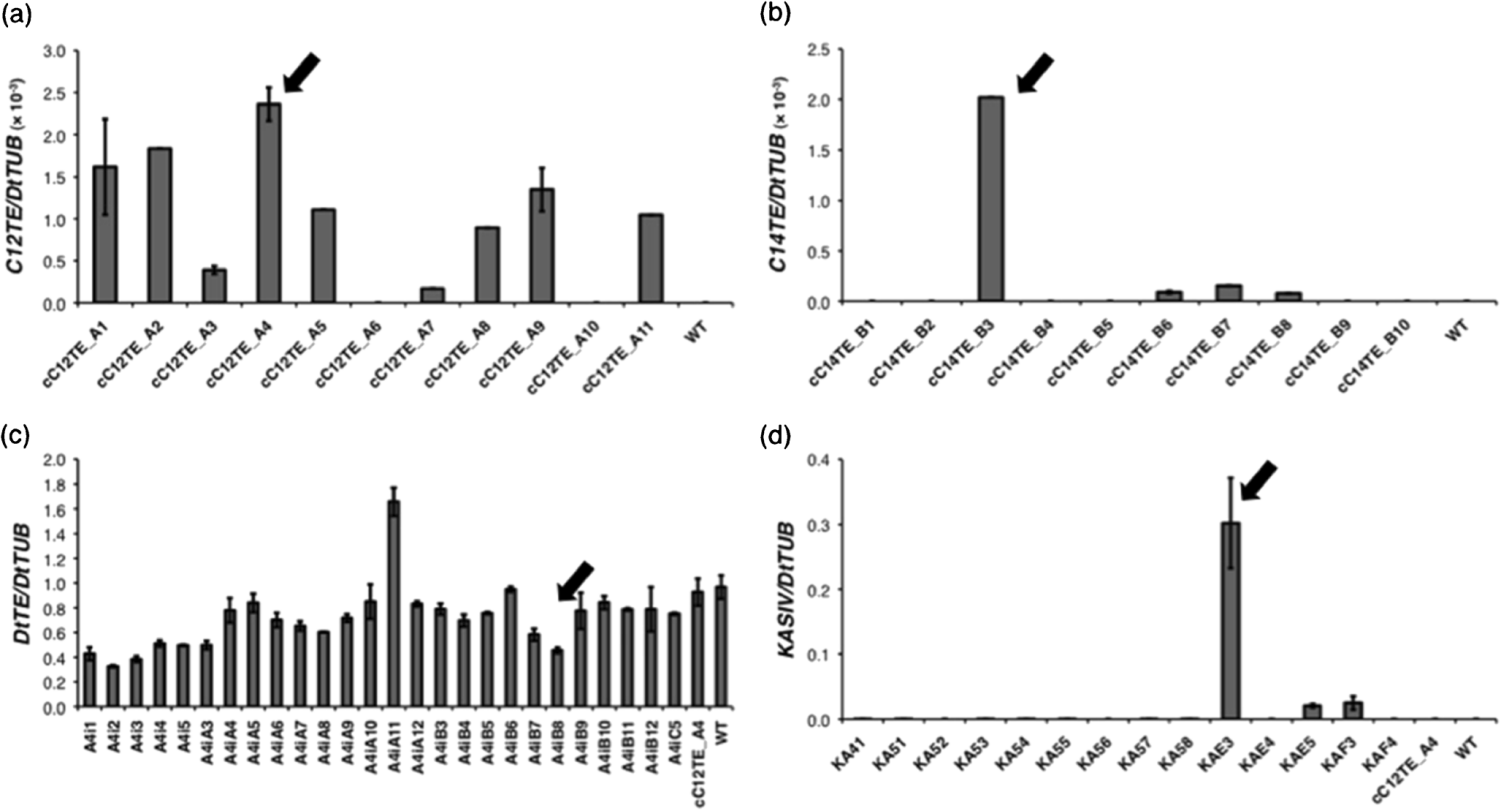
Transcript abundance analysis of native and transgenic *D. tertiolecta* strains. Gene expression detected by real-time qPCR in strains **a** UcC12TE, **b** CcC14TE, **c** UcC12TE + DtTE KD, and **d** UcC12TE + ChKASIV. *Arrows* indicate the transgenic *D. tertiolecta* strains selected for further phenotypic characterization. Values are the average of three separate experiments, and *error bars* show the standard deviation

The primary aim of this study was to investigate whether the availability of fatty acyl-ACP substrate pool for TE activity is one factor that limits the accumulation of MCFAs in transgenic *D. tertiolecta* strains. We hypothesize that (1) there is a competition between exogenous and endogenous TEs and (2) KAS is capable of distributing different chain lengths of fatty acyl-ACPs. We constructed two generations of *D. tertiolecta* strains for MCFA production (Fig. 1). All of the strains were carefully segregated to ensure their genotypic and phenotypic purities. In the first-generation *D. tertiolecta* strains (UcC12TE and CcC14TE), we selected C12TE and C14TE, from *U. californica* and *C. camphora*, respectively, based on their abilities to produce MCFAs (Radakovits et al. 2011). These genes were cloned in the well-characterized pGreenII 0000 plasmid (Hellens et al. 2000) and were separately transformed into wild-type *D. tertiolecta*, under the selection of zeocin resistance. We also included the chloroplast transit peptide from DtSBPase to ensure that the transgenic TE protein was directed to the site of de novo FA synthesis in the chloroplast. Multiple independent zeocin^r^ colonies were obtained and screened by genomic PCR. Figure 2a, b shows the genomic PCR gel images of the successful amplification of partial fragments of C12TE and C14TE from some of the transformed strains. Specifically, a 0.5-kb amplified product was detected, using either C12TE or C14TE gene-specific primers. As expected, exogenous C12TE and C14TE were not detected in wild-type *D. tertiolecta*. C12TE and C14TE transcript levels of the transgenic strains were determined by real-time qPCR (Fig. 3a, b). All transgenic strains revealed varying transgene expression levels, which might possibly be due to epigenetic silencing effects of transgenes or different numbers/locations of integrated transgene copies (Cerutti et al. 1997; Wu-Scharf et al. 2000).

For the second-generation *D. tertiolecta* strain (UcC12TE + DtTE KD), we suppressed the expression of the *DtTE* gene by RNA interference (RNAi). This was performed to investigate the competitive nature of endogenous TE in a MCFA-producing strain of *D. tertiolecta*. An RNAi plasmid was transformed into mutant cC12TE_A4 *D. tertiolecta* (from UcC12TE background) under the selection of norflurazon resistance. Endogenous TE could not be completely knocked out because some LCFAs are required to synthesize essential membrane lipids (Chapman and Ohlrogge 2012). Several norflurazon^r^ colonies were isolated for genomic PCR screening. An expected 0.5-kb amplified product using gene-specific DtTE-AS sense primer and GUS anti-sense primer was obtained in the double C12TE overexpressed and DtTE knockdown mutants (Fig. 2c). The 26 positive transformants from genomic PCR results were analyzed for the relative *DtTE* gene expression levels by real-time qPCR. Out of the 26 norflurazon^r^ colonies, 8 showed reduced *DtTE* gene expression by about 50%, while 11 showed reduced *DtTE* gene expression by about 20% with respect to the mutant cC12TE_A4 and wild-type *D. tertiolecta* (Fig. 3c). This reduction of *DtTE* expression was, however, far from the previously reported *A. thaliana* FatB TE knockout mutant, which had displayed 150-fold lower in expression than that of control plant (Bonaventure et al. 2003). Therefore, UcC12TE + DtTE KD mutants were considered as strains deficient in *DtTE* gene expression but not a total loss of function.

For the second-generation of *D. tertiolecta* strain (UcC12TE + ChKASIV), to allow a fair comparison with the strain UcC12TE + DtTE KD, KASIV from *C. hookeriana* was transformed into mutant cC12TE_A4 *D. tertiolecta* (from UcC12TE background). Studies on seed oil profiles in the *Arabidopsis* plants overexpressing both *Cuphea* KASIV and FatB TE reported a reduction in the levels of C16:0 and an enhancement in the levels of C12:0 (Leonard et al. 1998). The overexpression of KASIV in a MCFA-producing strain of *D. tertiolecta* would allow us to examine whether the bottleneck of further MCFA accumulation lies in the control of a KAS enzyme to generate MCFA acyl-ACP substrates. Several norflurazon^r^ colonies were isolated, and the presence of KASIV was verified by genomic PCR, using the KASIV gene-specific primers (Fig. 2d). As expected, exogenous KASIV could not be detected in mutant cC12TE_A4 and wild-type *D. tertiolecta*. Additionally, we only confirmed the expression of the *KASIV* gene in one transgenic strain (Fig. 3d).

### Co-expression of C12TE and KASIV increased MCFA production by fourfold

**Fig. 4:**
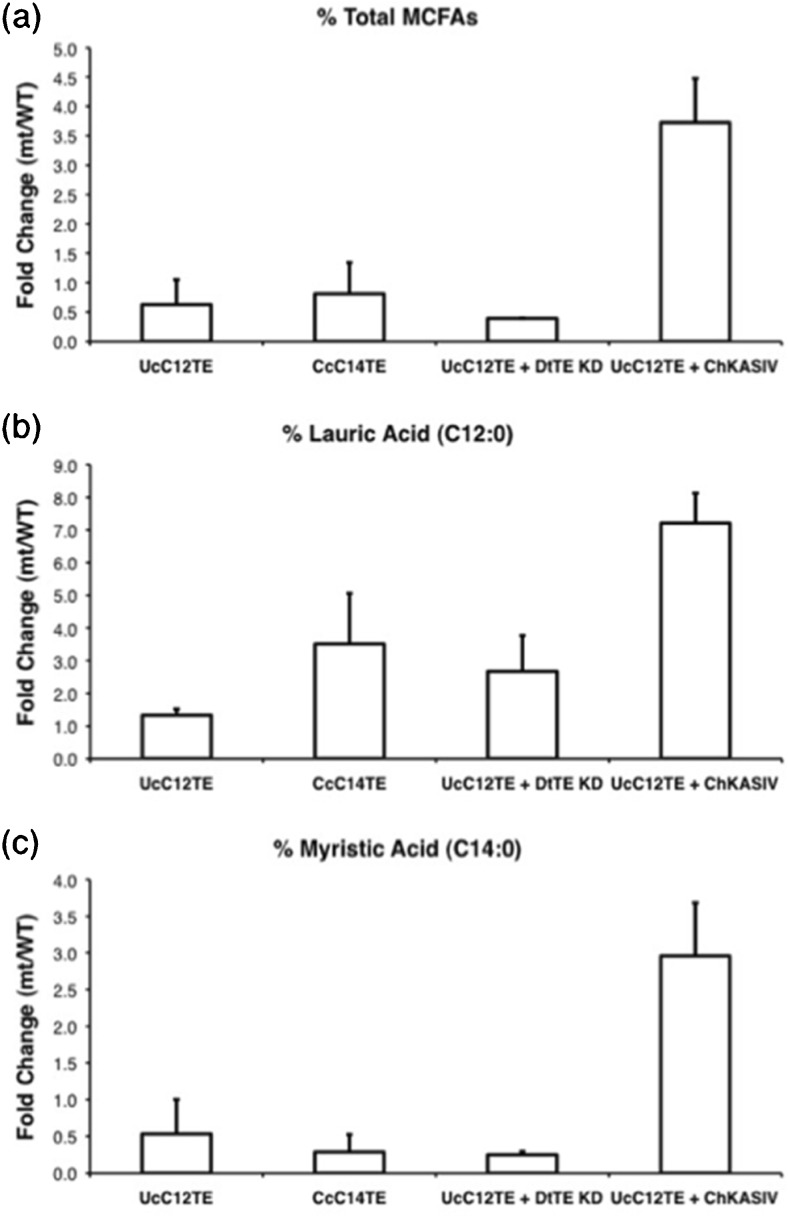
Enhanced MCFA production in transgenic *D. tertiolecta* strains. The increments in the **a** percentages of total MCFAs, **b** percentages of lauric acid (C12:0), and **c** percentages of myristic acid (C14:0) are expressed as fold changes (mutant/wild-type). Values are the average of three separate experiments, and *error bars* show the standard deviation. *UcC12TE U. californica* C12:0-biased FatB TE, *CcC14TE C. camphora* C14:0-biased FatB TE, *DtTE KD D. tertiolecta* TE knockdown, *ChKASIV C. hookeriana* MCFA-specific KAS

Production of storage lipids in *D. tertiolecta* is usually low during the exponential growth phase and increases during the stationary phase (Tan et al. 2016). Therefore, all strains were harvested at the stationary phase for higher detection sensitivity, and MCFA levels were determined by GC-MS. Increments of MCFA amounts in transgenic *D. tertiolecta* strains were then represented in fold changes with respect to the wild-type *D. tertiolecta* MCFA amounts (Fig. 4). Except for strain UcC12TE + DtTE KD, most genetic modifications had resulted in significant increments of MCFA production compared with the wild-type *D. tertiolecta*. In strain UcC12TE and CcC14TE, expression of single C12TE and C14TE, respectively, resulted in almost onefold increased total MCFA production (Fig. 4a), with strain Cc14TE giving the greatest response of fourfold increased C12:0 accumulation (Fig. 4b). Unexpectedly, in strain Cc14TE, there was barely an increase in C14:0 accumulation (Fig. 4c). MCFA production was further enhanced by co-expression of KASIV. In strain UcC12TE + ChKASIV, there was fourfold increased MCFA accumulation (Fig. 4a). Levels of C12:0 and C14:0 respectively increased by sevenfold and threefold (Fig. 4b, c).

We have successfully increased accumulation of MCFAs in those transgenic *D. tertiolecta* strains constructed by the single overexpression of C12TE or C14TE and also the double overexpression of C12TE and KASIV. However, our observations were in contrast to a previous report by Blatti and colleagues (2012). They did not observe a significant increase in the MCFA levels after introducing the C12:0-biased FatB TE from *U. californica* alone, because of the weak protein-protein interaction between the exogenous TE and the endogenous acyl-ACP. Our results showed that the single introduction of MCFA-specific TE (C12TE or C14TE) could actually increase the production of MCFAs. This disparity may be explained by the location of transgene deposition in their respective genomes and the method of MCFA analysis. Blatti and colleagues analyzed MCFA levels in transgenic *C. reinhardtii* strains during the exponential growth phase. On the other hand, we analyzed MCFA levels at the stationary phase to induce TAG production. Few studies of native *Chaetoceros* strain GSL56 (Gu et al. 2016), transgenic strains of *C. reinhardtii* (Inaba et al. 2017), and *P. tricornutum* (Radakovits et al. 2011) revealed that majority of MCFAs produced were preferentially incorporated into TAGs. It may be more effective to detect minor differences in MCFA amounts between the native and transgenic strains under our harvesting conditions. Nonetheless, the increment we observed was modest, and more works would be needed to further optimize the accumulation of MCFAs.

For our purpose of improving MCFA synthesis in *D. tertiolecta*, the double genetic modification approach, involving co-expression of C12TE and KASIV, performs better than the traditional approach of a single introduction of MCFA-specific TE and the silencing of endogenous DtTE. The progressive increases in C12:0 and C14:0 accumulation in strain UcC12TE + ChKASIV demonstrated that C12TE and KASIV enzymes both contribute to the regulation of FA chain lengths in *D. tertiolecta*. This approach can be employed to increase MCFA composition of the microalgae oil. Enhanced MCFA production in our double transgenic *D. tertiolecta* strain may be due to (1) enlargement of the MCFA acyl-ACP substrate pool by overexpressing KASIV (Dehesh et al. 1998), (2) improvement to an overall FA synthase rate that matches a TE activity (Dehesh et al. 2001), and/or (3) concentration of MCFA acyl-ACP substrate pool caused by KAS enzyme (Abbadi et al. 2000). On the other hand, the lack of significant DtTE knockdown impact on the production of MCFAs in transgenic *D. tertiolecta* strain suggested that endogenous DtTE was not a major controller of MCFA acyl-ACP substrate pool.

### Enhanced MCFA accumulation had negative impact on total lipid production in transgenic *D. tertiolecta* strains

**Fig. 5:**
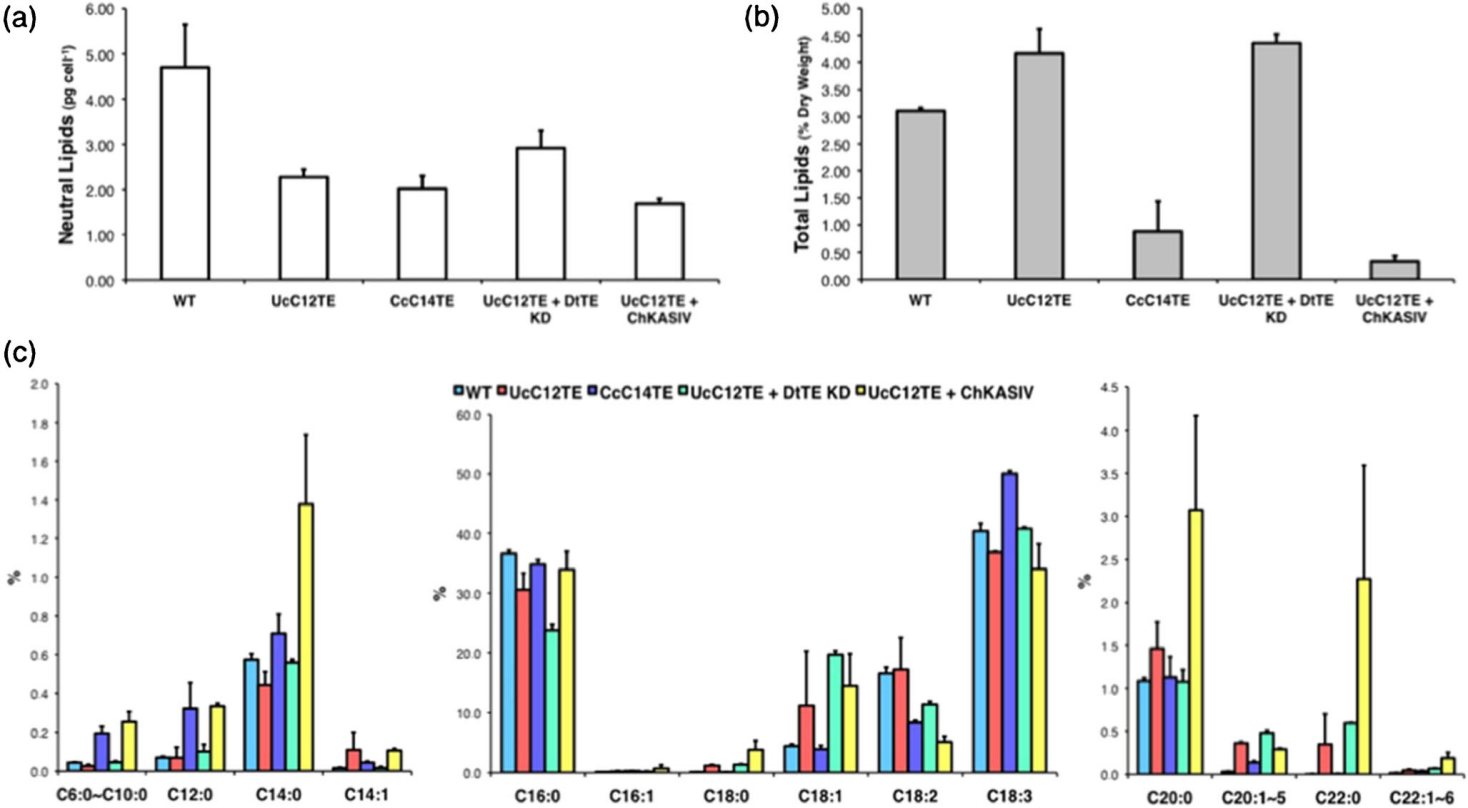
Quantitative analyses of neutral lipids, total lipids, and FA profiles. **a** Neutral lipids were estimated using Nile red assay. **b** Total lipids per dry weight were measured by GC-MS. **c** FA compositions were calculated based on weight percentages (%). Values are the average of three separate experiments, and *error bars* show the standard deviation. *UcC12TE U. californica* C12:0-biased FatB TE, *CcC14TE C. camphora* C14:0-biased FatB TE, *DtTE KD D. tertiolecta* TE knockdown, *ChKASIV C. hookeriana* MCFA-specific KAS

Microalgae synthesize and store different kinds of lipids in a single cell (Hu et al. 2008). In contrast to the conventional FA compositions of microalgae that contain LCFAs from C16 to C18, MCFAs (from C8 to C14) are generally classified to be unusual. It is possible that an increased production of MCFAs would potentially have deleterious effects on the cells. Nile red assay revealed that the mutants accumulated half the amounts of neutral lipids than wild-type *D. tertiolecta* (Fig. 5a). The decreased production of neutral lipids could be the result of decreased synthesis of total lipids. Two strains, namely CcC14TE and UcC12TE + ChKASIV, had displayed almost threefold reduction of total lipids (Fig. 5b). It is surprisingly to note that these strains were also the highest MCFA producers, in particular for C12:0 accumulation (Fig. 4b). The observation could suggest physiological responses of transgenic *D. tertiolecta* strains towards heightened levels of unusual MCFAs. Nonetheless, we also could not exclude that the decreased production of total lipids was due to secondary mutations.

Our observation here opposed to the previous reports of higher lipid production and free FA secretion in bacterial and cyanobacterial systems after the overexpression of MCFA-specific TEs (Voelker and Davies 1994; Lu et al. 2008). In those reports, the increment of total lipid production was dependent on the simultaneous overexpression of TEs with the inactivation of FA catabolic pathways. Furthermore, eukaryotic microalgae (*D. tertiolecta*) and bacteria/cyanobacteria are innately different, given that eukaryotic microalgae are capable to store free FAs in the form of storage TAGs. This could explain the reason for not able to detect any free FA secretion from *D. tertiolecta* cells, and this was also supported by other FA secretion works done on eukaryotic microalgae (Radakovits et al. 2011; Tan and Lee 2017).

Some differences in FA profiles of transgenic *D. tertiolecta* strains were also observed (Fig. 5c). Transgenic *D. tertiolecta* strains exhibited an increment in very long-chain-length FAs (vLCFAs) and a slight reduction in C16:0 and C18:3 that are the major FA species types in *D. tertiolecta* (Chen et al. 2011). This could be explained by the additional TEs in the transgenic strains, which have conferred substrate preference for saturated acyl-ACPs. Nevertheless, increased accumulation of vLCFAs may also reflect a change in the transgenic strain membrane properties. The mechanism that edits out unusual MCFAs from membrane lipids and channels them to proper storage lipids is imperative for unicellular microalgae. It is believed that MCFA would affect the structural integrity of membranes and have detrimental effects on the cells (Millar et al. 2000). Therefore, mechanisms that regulate MCFA accumulation are not solely govern by the pool of MCFA acyl-ACP substrates but also how the transgenic strains cope with the unusual MCFA buildup in the cells.

## Conclusion

Our results showed that the double-genetic engineering strategy of employing chain-length specific TE and KAS significantly enhanced MCFA composition of *D. tertiolecta* oil. This study illustrates that enlarging MCFA acyl-ACP substrate pool is one feasible approach that may be useful in conjunction to other strategies to sustainably produce good quality of microalgae-based biodiesel containing high levels of MCFAs. While transgenic TE and KAS are able to redirect FA synthesis from long to medium chain lengths, the increases in yield are still not as high as those achieved in plants and bacteria. Future efforts to understand microalgal FA homeostasis are necessary, whether targeting MCFA productivity or other unusual FA chain lengths.
